# BaHf_0.05_Ti_0.95_O_3_ Ceramics from Sol–Gel and Solid-State Processes: Application to the Modelling of Piezoelectric Energy Harvesters

**DOI:** 10.3390/ma17071508

**Published:** 2024-03-26

**Authors:** Damien Brault, Philippe Boy, Franck Levassort, Guylaine Poulin-Vittrant, Claire Bantignies, Thien Hoang, Maxime Bavencoffe

**Affiliations:** 1 GREMAN UMR 7347, INSA–CVL, University of Tours, CNRS, 41000 Blois, France; damien.brault@univ-tours.fr (D.B.); franck.levassort@univ-tours.fr (F.L.); guylaine.poulin-vittrant@univ-tours.fr (G.P.-V.); 2 CEA/Le Ripault, BP16, 37260 Monts, France; philippe.boy@cea.fr; 3 Innovation Department, VERMON S.A., 37000 Tours, France; c.bantignies@vermon.com (C.B.); t.hoang@vermon.com (T.H.)

**Keywords:** piezoelectric materials, lead-free materials, thin films, materials and devices for energy harvesting, electrical impedance, finite element modelling

## Abstract

A typical piezoelectric energy harvester is a bimorph cantilever with two layers of piezoelectric material on both sides of a flexible substrate. Piezoelectric layers of lead-based materials, typically lead zirconate titanate, have been mainly used due to their outstanding piezoelectric properties. However, due to lead toxicity and environmental problems, there is a need to replace them with environmentally benign materials. Here, our main efforts were focused on the preparation of hafnium-doped barium titanate (BaHf_x_Ti_1−x_O_3_; BHT) sol–gel materials. The original process developed makes it possible to obtain a highly concentrated sol without strong organic complexing agents. Sol aging and concentration can be controlled to obtain a time-stable sol for a few months at room temperature, with desired viscosity and colloidal sizes. Densified bulk materials obtained from this optimized sol are compared with a solid-state synthesis, and both show good electromechanical properties: their thickness coupling factor *k_t_* values are around 53% and 47%, respectively, and their converse piezoelectric coefficient $d_{33}^{*}$ values are around 420 and 330 pm/V, respectively. According to the electromechanical properties, the theoretical behavior in a bimorph configuration can be simulated to predict the resonance and anti-resonance frequencies and the corresponding output power values to help to design the final device. In the present case, the bimorph configuration based on BHT sol–gel material is designed to harvest ambient vibrations at low frequency (<200 Hz). It gives a maximum normalized volumetric power density of 0.03 µW/mm^3^/Hz/g^2^ at 154 Hz under an acceleration of 0.05 m/s^2^.

## 1. Introduction

Nowadays, energy harvesters, like piezoelectric energy harvesters (PEH), offer the possibility of directly converting an ambient vibration into electrical energy. A typical PEH structure consists of a bimorph clamped cantilever composed of two piezoelectric layers on both sides of a flexible substrate. Bimorph cantilever PEH requires the development of few hundred micrometer-thick piezoelectric layers. That latter can be achieved by thinning bulk ceramics [1,2], electrophoretic deposition (EPD) [3], or by composite sol–gel deposition [4].

Environmental and toxicity considerations about lead-based materials have led to the need to replace them. Among all the possible PZT substitutes studied, these can be gathered in three main families, all derived from the perovskite structure, namely sodium or potassium niobate (K/Na NbO_3_), bismuth titanate (BiTiO_3_), or barium titanate (BaTiO_3_) [5]. This latter possesses inner properties in its pristine form close to PZT. Moreover, this material is widely studied to understand the fundamentals of piezoelectricity or ferroelectricity, and its chemical modifications are a common means of tuning and improving its piezoelectric properties [6]. However, its low Curie temperature of around 120 °C limits its application to room-temperature scenarios [7,8].

This work focuses on the study of the piezoelectric properties of BaHf_0.05_Ti_0.95_O_3_ samples (referenced as BHT5), as this composition shows interesting piezoelectric properties with a piezoelectric coefficient $d_{33}\approx 300$ pC/V, a coupling coefficient in 33 mode $k_{33}=57\%$, and a coupling coefficient in thickness mode $k_{t}=47\%$ [9,10]. As demonstrated by Elorika et al. [11], between 3 and 8% in hafnium substitution, BaHf_x_Ti_1−x_O_3_ has a composition of mixed orthorhombic, tetragonal, and rhombohedral phases that could enhance its properties. They also demonstrated that these compositions have interesting properties for high-energy capacitors, as well as for optoelectronics applications, as their optical bandgap widens with Hf content, from 2.8 to 2.9 eV.

The process of producing thick films for PEH structure involves the development of a liquid chemical route, such as the sol–gel process, that enables the deposition of active layers on a substrate. As far as the liquid synthesis of hafnium-based barium titanate is concerned, few studies deal with BHT materials, but we can cite Fernandez et al. [12] who recently developed a nitrate-based sol–gel route to synthetize Ba_0.85_Ca_0.15_Hf_x_Ti_1−x_O_3_ (x = 0 to 0.15) and studied their optical properties in thin films. With this in mind, we have developed a sol–gel route of hafnium-doped barium titanate [10] and demonstrated the piezoelectric potential obtained on bulk material by this route. We have shown that this sol is stable over time and that its viscosity can be controlled. These properties make it possible to develop films with a thickness of around 10 µm [4], which are needed to produce PEH structures. Before developing thick films, we verified the viability of the BHT5 materials by numerical studies based on experimental values obtained on bulk materials. 

In the present study, the material was prepared either by a conventional solid-state synthesis or by a developed sol–gel one [10]. These two syntheses were used to produce pellets, whose structural properties were investigated. After poling, we measured the complex electrical impedance to deduce the electromechanical properties of these pellets. The corresponding piezoelectric parameters of the sol–gel route were compared with those of the solid-state in order to validate our synthesis route. Finally, these parameters were used to perform numerical studies to determine the working frequencies and harvested power values of these BHT-based PEH devices and compared with the performance obtained with a commercial PZT material.

## 2. Materials and Methods

### 2.1. Synthesis of BHT5 Samples

#### 2.1.1. Sol–Gel Samples

The BHT sol–gel route used for the preparation of samples has been adapted from [4,13] and previously published. Indeed, the sol–gel synthesis of BaHf_0.05_Ti_0.95_O_3_ (BHT5–SG) is realized according to the following route: barium acetate (Ba(CH_3_COO)_2_, Sigma Aldrich, 99%, Merck KGaA, Saint-Louis, MO, USA) is mixed with concentration of 0.5 M, in hot ethylene glycol (EG, Sigma Aldrich, 99%, Merck KGaA, Saint-Louis, MO, USA) at 70 °C, to create a first solution. A second solution is made by dissolving titanium (Ti(OiPr)_4_, Merk ≥ 98%, Merck KGaA, Saint-Louis, MO, USA) and hafnium isopropoxide (Hf(OiPr)_4_, Alpha Aeser, 99.9%, Merck KGaA, Saint-Louis, MO, USA) under an inert atmosphere (Ar) in isopropanol (iPrOH, Sigma Aldrich, 99.5%, Merck KGaA, Saint-Louis, MO, USA) and under vigorous stirring for 1 h. The two solutions are mixed, and isopropoxide molecules are removed by distillation at a temperature of 160 °C. Then, glacial acetic acid (AA, Sigma Aldrich, 99.7%, Merck KGaA, Saint-Louis, MO, USA) is added at 100 °C to stabilize the complex mixture and left to cool and to finally obtain a nearly 0.8 M solution. Then, the evaporation of solvents (water and alcohol) is ensured by heating the sol at 70 °C for 3 days. Finally, the obtained dried gel is grinded in a mortar and calcinated at a temperature of 1200 °C for 2 h to obtain final powder.

#### 2.1.2. Solid-State Samples

The solid-state synthesis of BaHf_0.05_Ti_0.95_O_3_ (BHT5–SS) has been realized following Yin’s route [14] using BaCO_3_ (Acros Organics, 99+%, Thermo Fischer Scientific, Waltham, MA, USA), TiO_2_ (Fluka, 99+%, Merck KGaA, Saint-Louis, MO, USA) and HfO_2_ (Alfa Aesar, 99+%, Merck KGaA, Saint-Louis, MO, USA). Typically, powders are weighed according to their stoichiometries. The mixture is poured into an yttrium–zirconia jar together with 20 g of zirconia balls and 25 mL of deionized water and planetary milled at 400 rpm for 2 h in RETSCH S100 (RETSCH GmbH, Haan, Germany). After drying, the obtained powder is calcinated at 1000 °C for 2 h in the air.

The powders (either prepared by the sol–gel or solid-state routes) were mixed with P.V.A. (2.5% wt., Sigma Aldrich, 99%, Merck KGaA, Saint-Louis, MO, USA) and pressed uniaxially at 80 MPa to obtain 30 mm diameter and 1 mm thick pellets. Pellets are sintered at 1500 °C in the air for 6 h for the solid-state route and 12 h for the sol–gel route. After their sintering, pellet densities were determined using geometrical measurements, and the theorical densities were estimated to be 6140 kg/m^3^ for BHT5 and 7950 kg/m^3^ for lead-based materials (later called NAVY II and NAVY III).

### 2.2. Characterizations

The crystallographic properties of the prepared BHT powders were analyzed with a D8 Brucker Advance diffractometer (Brucker, Billerica, MA, USA) with Cu wavelength (1.541 Å).

Measurement of strain loops upon electric field were performed at room temperature using 10 Hertz (Hz) triangular waves of 1 kV/mm amplitude on AixPES–PSHU (AixACCT system GmbH, Aachen, Germany) combined with a Series SP 120/2000 interferometer laser (SIOS meβtechnick GmbH, Ilmenau, Germany). Polarization of the samples was also performed on AixPES–PSHU at a temperature of 60 °C for 5 min under field cooling conditions at 1 kV/mm.

The samples’ impedances measurement was performed on a calibrated Agilent 4294A (Agilent technologies Inc., Santa Clara, CA, USA) around the radial and thickness resonances modes. The obtained impedance curves were fitted with Krimoltz–Leedom–Matthaei (KLM) model to determine their electromechanical characteristics [15,16], and using an equivalent electrical circuit model where dielectric and mechanical losses are introduced. Elastic stiffness and compliance coefficients ( $C_{33}^{D}$ and $S_{11}^{E}$), as well as transverse and radial ( $v_{t}$ and $v_{p}$) acoustic wave celerities, were deduced from the measurement of the anti-resonant frequency ( $f_{0}^{t}$ at the maximum value of the impedance in the thickness resonant mode); $f_{0}^{p}$ at the maximum value of the impedance in the radial resonant mode), as follows:

(1) $$\left\{\begin{array}{c} C_{33}^{D}={\left(2\cdot t\cdot f_{0}^{t}\right)}^{2}\\ v_{t}=\sqrt{\frac{C_{33}^{D}}{\rho }}\\ S_{11}^{E}=\;{\left(\frac{\eta }{2\cdot \pi \cdot r\cdot f_{0}^{p}}\right)}^{2}\cdot \frac{1}{\left(1-\sigma^{2}\right)\cdot \rho }\\ v_{p}=\frac{1}{\sqrt{S_{11}^{E}\cdot \left(1-\sigma^{2}\right)\cdot \rho }} \end{array}\right.$$

where *t* and *r* are, respectively, the thickness and radius of the sample disk, and *ρ* its density (determined by geometrical and weight measurements); *η* is the first root of $\left(1+\sigma \right)\cdot J_{1}^{\left(z\right)}=n\cdot J_{0}\left(z\right)$ ( $J_{n}\left(z\right)$, being the Bessel’s functions of the first kind and nth order) and $\sigma =-S_{12}^{E}/S_{11}^{E},$ the Poisson’s coefficient. The two coefficients *η* and *σ* can be approximated accordingly to a polynomial function depending on the ratio $\alpha =f_{0}^{p}/f_{1}^{p}$ of the fundamental $\left(f_{0}^{p}\right)$ and the first overtone ( $f_{1}^{p}$) of the radial resonant peaks [17], as follows:

(2) $$\left\{\begin{array}{c} \sigma \approx 97.53-126.92\cdot \alpha +63.40\cdot \alpha^{2}-14.34\cdot \alpha^{3}+1.23\cdot \alpha^{4}\\ \eta \approx 11.29-7.64\cdot \alpha +2.14\cdot \alpha^{2}-0.22\cdot \alpha^{3} \end{array}\right.$$

A fitting process was performed on the complex experimental electrical impedance (*Z*) for the thickness and admittance (*Y*) for the radial resonant mode, according to the following standard equations [16]:

(3) $$\left\{\begin{array}{c} Thickness\;mode\;⇨\;Z\left(\omega \right)=\frac{t}{i\cdot \omega \cdot \varepsilon_{33}^{s}\pi r^{2}}\cdot \left(1-k_{t}^{2}\frac{tan(\omega \cdot t/2v_{t})}{\omega \cdot t/2v_{t}}\right)\\ Radial\;mode\;⇨\;Y\left(\omega \right)=\frac{t}{i\cdot \omega \cdot \varepsilon_{33}^{T}\pi r^{2}}\cdot \left(\frac{k_{p}^{2}}{1-k_{p}^{2}}\cdot \frac{1+\sigma }{1-\sigma -\Gamma \left(\omega r|/|v_{p}\right)-1}\right) \end{array}\right.$$

where ω=2⋅π⋅f with *f* is the frequency; $\Gamma \left(\varphi \right)=\varphi \cdot J_{0}\left(\varphi \right)/J_{1}\left(\varphi \right)$, with $\varphi =\omega r/v_{p}$; their coupling factors in thickness and radial mode are defined as follows [16]:

(4) $$\left\{\begin{array}{c} Thickness\;mode\;⇨\;k_{t}\;=\;\frac{e_{33}}{\;\sqrt{\varepsilon_{33}^{S}C_{33}^{D}}}\\ Radial\;mode\;⇨\;k_{p}\;=\;\frac{\;\sqrt{2}\cdot d_{13}}{\sqrt{S_{11}^{E}\left(1-\sigma \right)\varepsilon_{33}^{T}}} \end{array}\right.$$

where $e_{33}$ is the piezoelectric charge coefficient and $d_{13}$ the piezoelectric strain constant.

The losses part of the dielectric constant ( $\varepsilon_{33}\equiv \varepsilon_{33}\cdot (1-i\cdot \tan\left(\delta_{e}\right))$ at a constant strain and at constant stress ( $\varepsilon_{33}^{S},\;\varepsilon_{33}^{T}$) were deduced from the ratio of the imaginary and real part of *Z*. Mechanical losses $\left(C_{33}^{D}\equiv C_{33}^{D}\cdot (1+i\cdot \tan\left(\delta_{m}\right)\right)$ or $S_{11}^{E}\equiv \;S_{11}^{E}\cdot (1-i\cdot \tan\left(\delta_{m}\right)))$ were deduced from resonant and anti-resonant frequencies of the susceptance [18].

Finally, 8 samples, resulting from the solid-state route and 2 samples from the sol–gel route were characterized by this method.

## 3. Results and Discussions

### 3.1. Structural Characterizations

**Figure 1:**
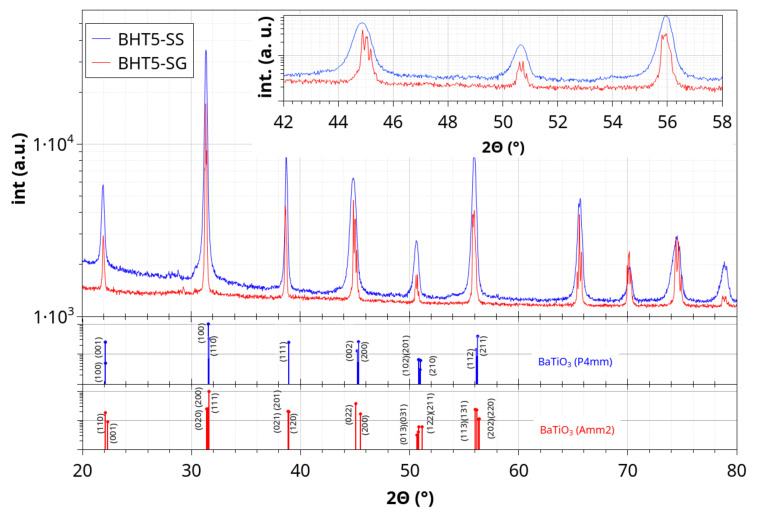
X-ray diffraction of BHT5 sintered pellets prepared from solid-state (SS, blue line) and sol–gel (SG, red line) routes, and tetragonal (P4mm, COD 1507756, blue vertical lines) and orthorhombic (Amm2, COD 9015715, red vertical lines) BaTiO_3_ patterns.

The X-ray diffraction patterns of BHT5 pellets prepared via solid-state (BHT5–SS) and sol–gel (BHT5–SG) powders are presented in Figure 1.

Samples structures coincide with a tetragonal barium titanate-like pattern (BaTiO_3_ P4mm, Crystallography Open Data—COD—1507756) for the solid-state route and orthorhombic structure for the sol–gel route (Amm2, COD 9015715). The hkl lines shown in Figure 1 correspond to pure BaTiO_3_ structures, and the hafnium substitution could induce an increase in the lattice parameter, as hafnium is bigger than titanium, so that the BHT5 diffractograms shown exhibit shifts compared to pure BaTiO_3_. One may expect that the difference in structure between the samples comes from the different processing route used. The liquid media used in the sol–gel route can induce a better incorporation of the hafnium into the lattice, and the different times and temperatures used for calcination and sintering can lead to a higher orthorhombicity in the lattice [14,19].

### 3.2. P–Ev and s–Ev Loops Characterizations

**Figure 2:**
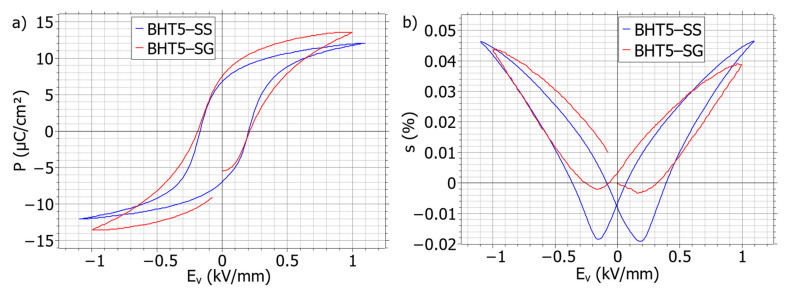
Polarization (**a**) and strain (**b**) field loops at 1 kV/mm of BHT5 sintered pellets prepared from solid-state (BHT5–SS, blue line) and sol–gel (BHT5–SG, red line) routes.

Characterizations of polarization (*P*) and induced strain (*s*) versus applied electric field (*E_v_*) are depicted for both BHT5–SS and BHT5–SG in Figure 2.

Polarizations (Figure 2a) of both samples display a comparable behavior against the electric field; the coercive fields have been deduced to be 210 ± 20 V/mm for the sol–gel processed samples and 190 ± 30 V/mm for the solid-states ones. As the plotted polarizations in Figure 2 have been taken under saturated conditions by applying 5 times these coercive fields, under these conditions, the two samples have close saturated polarizations, respectively, of 12 and 13.5 µC/cm^2^, and agree with the reported values in the literature [14].

The induced strain (Figure 2b) over the field displays the two typical “butterfly” figure forms of bipolar loops. However, the two samples show different strain behaviors under the electric field.

The two samples show a similar maximum strain around 0.045% at 1 kV/mm, as displayed in Figure 2. The first observed dissimilarity lies in a more pronounced negative part of the strain (*S_neg_*) in BHT5–SS in comparison with BHT5–SG. There may exist three main contributions to the strain: the first comes from the electrostriction effect but is only relevant at a low electric field; the second is from the piezoelectric effect; the last is from 180° and non-180° switching domains [20,21]. The depth of *S_neg_* should depend mostly on the non-180° switching domains [22,23,24] and could affect the apparent piezoelectric coefficient $d_{33}^{*}$, with this one being evaluated to be about 420 pm/V for BHT5–SS at 1 kV/mm versus 330 pm/V for BHT5–SG. The dynamics of domain switching and their impact on the internal piezoelectric or ferroelectric properties of BHT5 are still under investigation to explain these differences or similarities.

Again, the deduced $d_{33}^{*}$ of the two samples possess values comparable to the previously reported ones [14,25].

We can also note that both strain and polarization cycles for the sol–gel present a little asymmetry, as the polarization is shifted to the left by 50 V/mm and its maximum strain in the left branch is higher than the right branch with a difference of 0.01%. In comparison, the solid-state processed sample is more symmetrical. This may be caused by the presence of some defected dipoles (such as oxygen vacancies) due to the longer dwelling time used for the sol–gel sintering in comparison to the solid-state process [26,27]. 

### 3.3. Impedance Measurements

**Figure 3:**
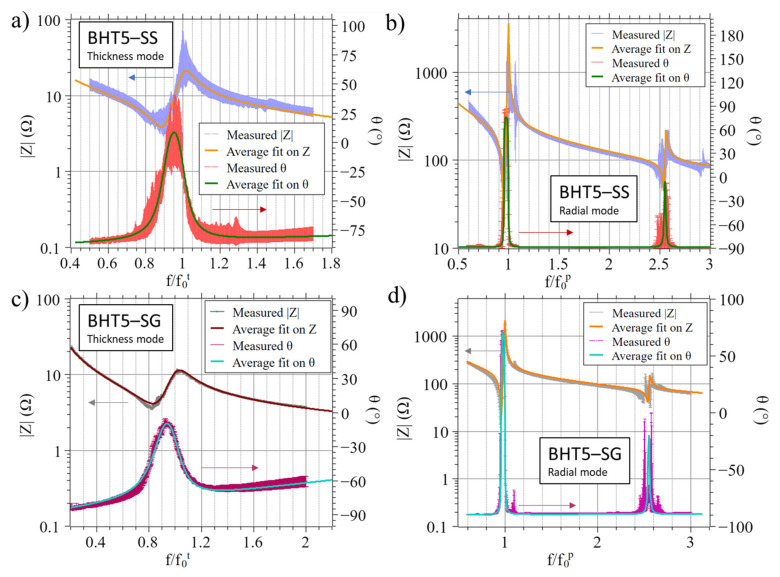
Impedance modulus (|*Z*|) and phase (*θ*) spectra as a function of the normalized frequencies (*f*/*f*_0_*^t^* and *f*/*f*_0_*^p^*) measured on BHT5–SS (|*Z*|): blue line; *θ* red line; average of 8 samples) and BHT5–SG (|*Z*|): grey line; *θ* purple line; average of 2 samples); (**a**,**c**) show the thickness resonance around its fundamental frequency (*f*/*f*_0_*^t^*), and (**b**,**d**) show the radial resonances around the fundamental *f*/*f*_0_*^p^* and first harmonic *f*/*f*_1_*^p^*; solid lines represent the average of spectra calculated using the KLM model, and arrows link curves to the corresponding *y*-axis.

The impedance measurements of BHT5–SS and BHT5–SG are depicted in Figure 3. The piezoelectric and electromechanical properties of BHT5–SS and BHT5–SG deduced from the impedance measurements (Figure 3) are presented in Table 1. The 8 BHT5–SS and 2 BHT–SG samples were averaged to retrieve mean values, with their relative dispersion being around 5% of the average values. The highest observed deviations, reaching 30% of the average values, are due to the dispersion of electrical and mechanical losses. As a result of these relative deviations in dielectric and electromechanical properties (including losses) observed in all samples (eight samples in the case of BHT5–SS and two samples for BHT5–SG), the impedance spectra presented in Figure 3 show certain dispersions.

The frequencies have been normalized by their respective anti-resonance frequencies, specifically $f_{0}^{t}$ and $f_{0}^{p}$.

### 3.4. Partial Tensors Reconstruction

**Figure 4:**
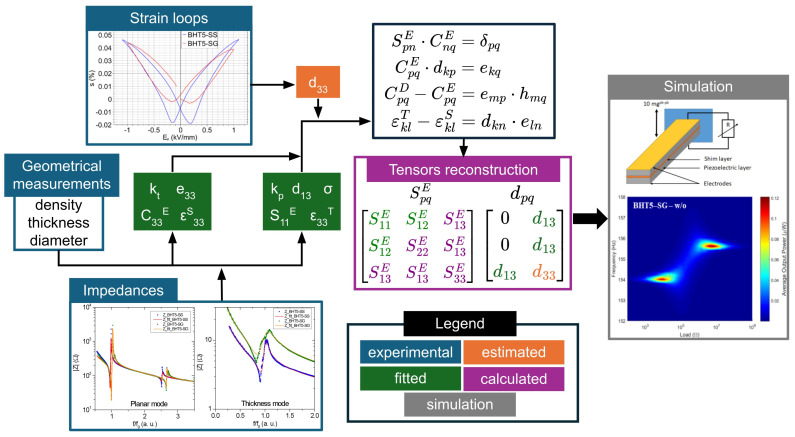
Scheme of the methodology used to determine the mechanical and piezoelectric tensors for finite element simulation.

To perform numerical simulations via finite element modeling, and to evaluate the harvesting performance of the simulated devices, we need access to the mechanical and piezoelectric tensors of the materials. The overall methodology used is depicted in Figure 4.

The calculations of the mechanical and piezoelectric tensors are based on experimental values (Figure 4, blue boxes). First, we can evaluate the $d_{33}$ piezoelectric coefficient of the materials (Figure 4, orange box). To achieve this, the coupling coefficient $d_{33}^{*}$ was measured by interferometry, taking the maximum positive strain divided by the applied field (Figure 2b). However, this method is not the most accurate for estimating this coefficient: as recommended in [32], it should be measured at a low field strength on poled materials. This interferometric measurement method is frequently used in the literature as a simple means of evaluating electromechanical properties as a function of substitution or grain size [33,34,35], or to assess the different processes or contributions involved in strain measurements at high electric fields [36,37]. Considering that the converse $d_{33}^{*}$ obtained by interferometry measurements can be overestimated by 10–40% compared to $d_{33}$ [34,38], a partial reconstruction of the piezoelectric matrix has been carried out using 70% of the measured $d_{33}^{*}$ (Table 1), resulting in an overestimation of 40% of the real $d_{33}$. We used this particular factor between $d_{33}$ and $d_{33}^{*}$ on the basis of global observations and comparisons between strain-field measurements and the Berlincourt method in comparable materials, such as BaTiO_3_ [39], BaCaTiHfO_3_ [40] or BaSrTiHfO_3_ [35], where the $d_{33}^{*}$ values obtained by strain-field measurements (at a high field of 10 to 15 times their respective coercive fields) and interferometry are around 30% higher than those measured by the Berlincourt method. Using this methodology, the estimated $d_{33}$ values (Table 1) are 300 pC/N for BHT5–SS and 263 pC/N for BHT5–SG. These values fall within the typical range of $d_{33}$ observed using the Berlincourt method in this composition [9,14,25]. Secondly, with the impedance measurements (Figure 3) and the Krimoltz–Leedom–Matthaei model (Equations (3) and (4)), we can recover the electromechanical constants $\varepsilon_{33}^{T;S},\;e_{33},\;C_{33}^{E;D},\;S_{11}^{E;D},\;\sigma \;$, and $d_{13}$ using a fitting process (Figure 4, green boxes). Finally, with these values and the evaluated $d_{33}$, we performed a partial reconstitution of the piezoelectric matrices (Figure 4, pink box), using the following standard equations [16,41] to calculate the $S_{13}^{E}$ and $S_{33}^{E}$ coefficients (see Table 1):

(5a)SpnE⋅CnqE=δpq(5b)ekq=CpqE⋅dkp(5c)CpqD−CpqE=emp⋅hmq(5d)εklT−εklS=dkn⋅eln

where $C_{pq}^{D}$ and $C_{pq}^{E}$ represent, respectively, the elastic stiffness constants, at constant electric displacement (*D*) and constant electric field (*E*), while $S_{pn}^{E}$ is the elastic compliance at constant electric field. The piezoelectric strain constant is represented by $d_{kp}$, $e_{kq}$ is the piezoelectric stress constant, and $h_{mq}=e_{mq}/\varepsilon_{kl}$ is the piezoelectric stiffness constant. The material permittivity constants are expressed at constant stress ( $\varepsilon_{kl}^{T})$ and constant strain ( $\varepsilon_{kl}^{S}$). One can note that we have used a Voight notation, so that, for example, Equation (5b) is actually $e_{kq}=\sum_{p}C_{pq}^{E}\cdot d_{kp}$ and $\delta_{pq}$ stands for the Kronecker delta.

The piezoelectric and electromechanical properties of BHT5–SS and BHT5–SG presented in Table 1 are compared with a reference PZT Navy type III material (subsequently called NAVY III), i.e., a Channel 5804 PZT ceramic (Channel Industries Ltd., Santa Barbara, CA, USA) [28,29] whose material parameters are close to those of the obtained BHT5 samples. Both the BHT5–SS and SG samples show promising electromechanical properties for applications in ultrasonic transduction, with $k_{t}$ values of up to 53% and $d_{33}$ values of up to 300 pC/N, in line with our previous study [10] and close to those of NAVYIII (Table 1). As for cantilever-based vibration energy harvesters, the following electromechanical coupling coefficient $k_{13}^{2}$ can be used to describe the efficiency of the piezoelectric material in converting mechanical energy into electrical energy [42]:

(6) $$k_{13}^{2}=\frac{d_{13}^{2}}{\varepsilon_{33}^{T}\;\cdot \;S_{11}^{E}}$$

The energy harvesting performance of a piezoelectric material can also be evaluated by the figure of merit (FOM), defined as follows [43]:

(7) $$\mathrm{FOM}=\frac{d_{13}^{2}}{\varepsilon_{33}^{T}}$$

For comparison purposes, we introduce a reference PZT Navy type II material (subsequently called NAVY II), i.e., a PZ27 ceramic (CTS|Ferroperm Piezoceramics, Kvistgård, DK) [30,31]. Regarding Curie temperature (see Table 1), BHT5 samples are suitable for energy harvesting applications at room temperature. Nevertheless, according to Table 2, leaded piezoelectric materials are almost three times more efficient regarding $k_{13}^{2}$ and are almost four times efficient in terms of FOM than BHT5 samples.

### 3.5. Simulations of BHT5-Based Harvesters

One of the main piezoelectric structures used in vibration energy harvesting is the bimorph, made of two piezoelectric layers separated with an inner elastic shim material [44]. Thinned-bulk piezoelectric energy harvesters show the capability to address lower frequencies (<200 Hz) corresponding to most ambient vibrations [45,46]. In this way, the samples obtained from BHT5–SS and BHT5–SG can be shaped and used to produce PEH devices [2,47].

**Figure 5:**
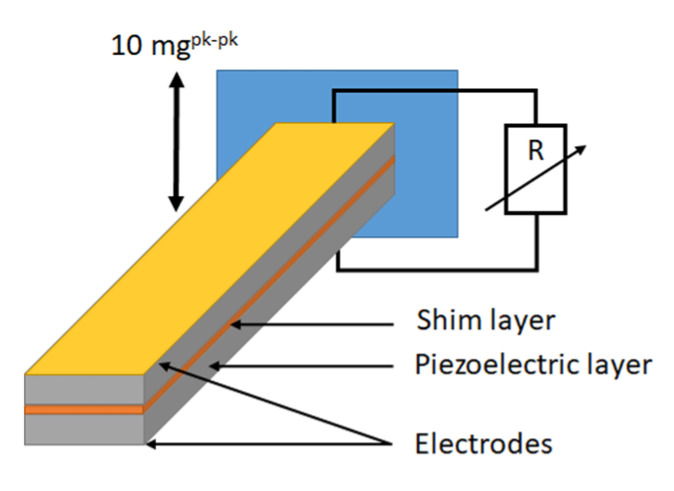
Schematic of the bimorph attached to the clamping fixture and connected to a resistive load (R).

Meanwhile, to understand and predict the behavior of such cantilever-based mechanical energy harvesters, numerical models have been developed using COMSOL Multiphysics^®^ FEA software (version 6.1) [48,49,50]. Here, frequency domain studies have been performed on a 3D FE model with respect to different resistive load values in clamped-free mechanical boundary conditions under an acceleration of 10 mg peak–peak (with 1 g = 9.80665 m/s^2^) to compute the average output electrical power of the considered samples (Figure 5).

In this continuity, bimorph structures of 4 mm × 39 mm area, 150/15/150 µm thick BHT5/brass/BHT5 layers, respectively, with gold electrodes on their upper and lower surfaces, are modelled with a hexahedral mesh of 936 elements (312 elements per layer; dimensions of a piezoelectric-layer element: 1 mm × 1 mm × 75 µm, dimensions of a brass-layer element: 1 mm × 1 mm × 7.5 µm). The clamping condition and acceleration are applied to a 4 mm × 3 mm area at one end of the structure.

The elastic, electric, and piezoelectric tensors, and mechanical and electric losses of the BHT5 layer are taken from Table 1. $S_{55}^{E}$, $d_{15}$ and $\varepsilon_{11}^{T}$ coefficients have a weak influence on the electrical response of the structure [38]. In addition, the material parameters of the BHT5 are close to those of the NAVYIII. For these two reasons, the values of the coefficients $S_{55}^{E}$, $d_{15}$, and $\varepsilon_{11}^{T}$ are identified with those of the NAVYIII reference material [41]. Comprehensively, the materials with high $Q_{m}$ and 1/tanδ exhibited good properties for energy transduction, probably due to reduced electrical and mechanical losses [51]. Once the mechanical and electrical losses of the BHT5 layer have been quantified, the influence of these on the response of the device can be appreciated. The material properties of the brass layer are obtained from the manufacturer data: density *ρ* = 8450 kg/m^3^, Young’s modulus *E* = 110 GPa, and Poisson’s ratio *σ* = 0.35.

**Figure 6:**
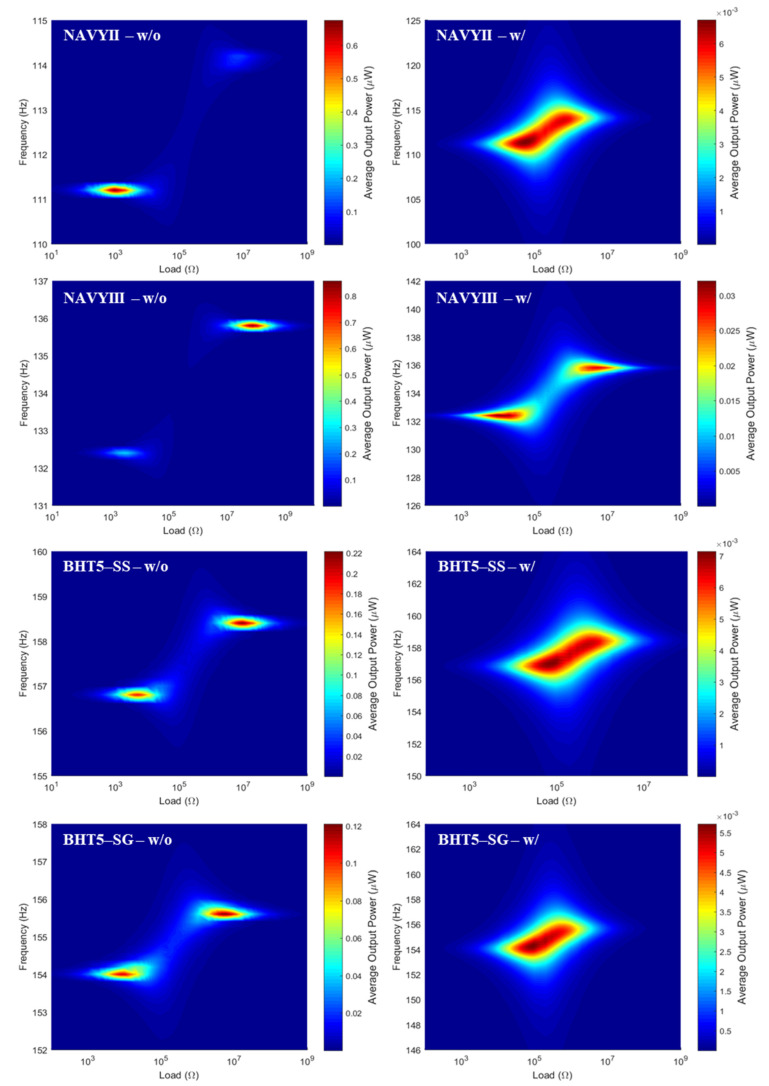
Distribution of the average output power as a function of the frequency and the value of the connected resistive load for different PEH devices according to the type of piezoelectric material used without (w/o) or with (w/) losses, under an acceleration of 10 mg^pk–pk^.

In the bimorph case, two peaks of harvested power are observed: one at the resonance frequency ( $F_{r}$) and the other at the anti-resonance frequency ( $F_{a}$). Figure 6 shows the distribution of the average output power as a function of both the frequency and the connected resistive load for different PEH devices according to the type of piezoelectric material, with (w/) or without (w/o) electrical and mechanical losses (modelled by $\delta_{e}$ and $\delta_{m}$ losses), under a peak–peak acceleration of 10 mg^pk–pk^.

Simulation results are summarized in Table 3 where $Z_{r}$, $P_{r}$, $BW_{r}$ are the optimal resistive load values, the average output power, and the half-height bandwidth at the resonance frequency $F_{r}$, respectively, and $Z_{a}$, $P_{a}$, $BW_{a}$ are the optimal resistive load value, the average output power, and the half-height bandwidth at the anti-resonance frequency $F_{a}$, respectively.

With constant geometric parameters, Figure 6 and Table 3 show that the simulated devices do not present peaks in the same frequency range. This is mainly due to the difference in material density. In fact, according to the analytical model proposed by Erturk et al. [52], the undamped natural frequency of the *r*th vibration mode in short circuit conditions is given by the following:

(8) $$F_{r}^{u}=1.875^{2}\sqrt{\frac{YI}{mL^{4}}}$$

where *YI* is the bending stiffness and *m* the mass, and *L* is the useful length of the bimorph. Table 4 provides the undamped natural frequencies of the first flexion mode obtained by Equation (8) and by FE simulation. As the density increases, the total mass of the device increases too, and $F_{r}^{u}$ decreases. Furthermore, the discrepancy between the results of the analytical and numerical models is less than 4% and is mainly due to the use of bulk parameters in simulations involving NAVYII and NAVYIII materials [35].

Even if the difference in material density mainly affects the frequency range of use of the simulated devices, their performances are of the same order of magnitude (between 0.1 µW and 1 µW) if the electrical and mechanical losses are not considered. However, leaded piezoelectric materials are still at least six times more efficient in terms of average output power.

When considering the electrical and mechanical losses, besides a slight change in the operating frequency range, the resonance and anti-resonance peaks become closer together and the performance gap between leaded and lead-free materials is reduced. Moreover, when losses are considered, the half-height bandwidth increases by a factor of almost 4 for NAVYIII, almost 11 for BHT5–SS and BHT5–SG, and up to 16 for NAVYII. The PEH frequency bandwidth is also an important criterion, due to the variability of the targeted mechanical vibration frequency [53]. Comparing Table 3 with Table 1, it can be seen that as the mechanical losses $\delta_{m}$ increase, so does the half-height bandwidth.

Since the energy-generation capacity varies from one device to another, a standardized criterion is needed to facilitate comparison; here, we use the normalized power density (NPD) [54], defined as follows:

(9) $$\mathrm{NPD}=\frac{Power\;[µW]}{Volume\;\left[{\mathrm{mm}}^{3}\right]\times Frequency\;\left[\mathrm{Hz}\right]\times {Acceleration}^{2}\;[g^{2}]}$$

The comparison between different PEH devices is shown in Table 5 [52,55,56,57,58,59,60,61,62,63,64,65,66,67].

**Figure 7:**
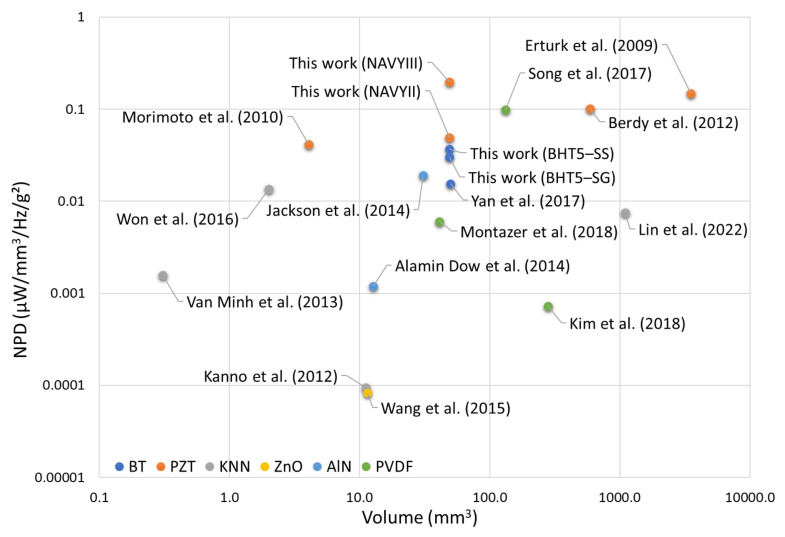
Normalized volumetric power density (NPD) as a function of the volume of the PEH devices [52,55,56,57,58,59,60,61,62,63,64,65,66,67].

**Figure 8:**
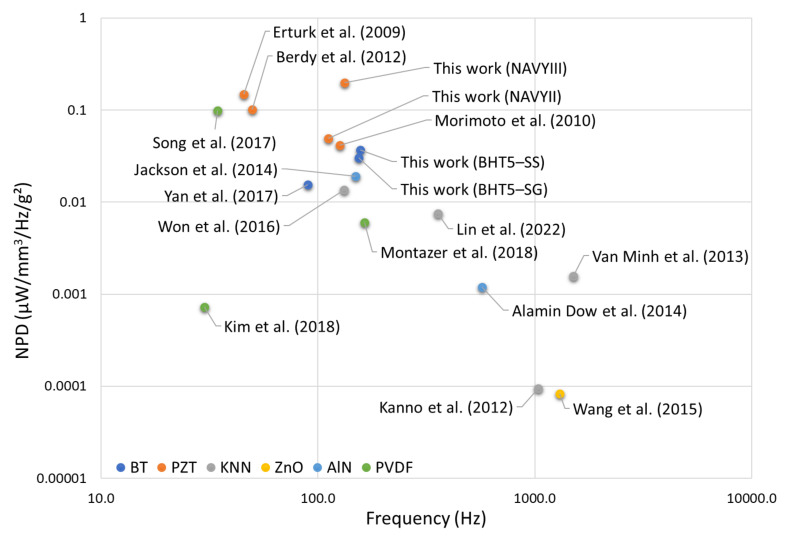
Normalized volumetric power density (NPD) as a function of the excitation frequency of the PEH devices [52,55,56,57,58,59,60,61,62,63,64,65,66,67].

Based on the data in Table 5, the plot of NPD as a function of volume of PEH devices is first presented in Figure 7. Similarly, Figure 8 plots NPD as a function of PEH device excitation frequency. In their configuration, the simulated BHT5-based harvesters present performances close to the NAVYII-based device, mainly due to the latter’s high losses. In addition, performances of the simulated NAVYIII-based device are superior to that of the NAVYII-based device, suggesting that hard piezoelectric property should be favored for a piezoelectric generator from a losses point of view [51].

## 4. Conclusions

The electromechanical properties of BHT5 samples prepared by solid-state and sol–gel route were investigated. The sol–gel process developed here enables bulk ceramics to be processed with electromechanical properties close to those of conventional solid-state processed ceramics, with their thickness coupling factor being around 53% and 47%, respectively, and their converse piezoelectric coefficient being around 420 and 330 pm/V, respectively. 

Using these parameters, we performed a partial reconstruction of their piezoelectric properties to predict the behavior of cantilever-based mechanical energy harvesters using a numerical model. These properties were also compared with those of reference PZT materials. 

The results show comparable piezoelectric properties between the PZT reference sample and the BHT5 samples. The main differences between them lie in a less densification of BHT5 (~85%) compared to PZT material (~95%). This leads to higher electrical and mechanical losses, which requires further optimization of the synthesis conditions.

When modelling bimorph-type piezoelectric energy harvesters based on BHT5 piezoelectric material, regardless of the material elaboration route (solid-state or sol–gel), the performances are equivalent in terms of average output power, operating frequency range, and half-height bandwidth at resonance and anti-resonance frequencies.

The lead-free PEH devices simulated here have a frequency bandwidth almost four times wider than that based on the reference hard PZT. This may facilitate frequency tuning, which is often required due to the variability of the mechanical source in a real application. Nevertheless, the reduction by a factor of six of the average output power implies the need to optimize the lead-free material, especially regarding the electrical and mechanical losses.

Even if further optimizations (of synthesis conditions, material, and device geometry) can be carried out, simulated BHT5-based devices show a Normalized volumetric Power Density close to that of other PZT-based PEH devices. Comparison between solid-state ceramics and sol–gel ceramics with similar properties has enabled us to validate our synthesis route and, in the case of sol–gel ceramics, to develop the manufacturing process for the thick films required for the PEH structure. This makes BHT5 a promising candidate to produce lead-free PEH devices designed to harvest low frequency ambient vibrations.

## Figures and Tables

**Table 1:** Electromechanical properties of BHT5–SS, BHT5–SG, NAVYIII [28,29] and NAVYII [30,31].

**Samples**	***ρ*** **(kg/m^3^)**	$f_{0}^{t}/f_{0}^{p}$ **(MHz/kHz)**	$\varepsilon_{33r}^{S}/\varepsilon_{33r}^{T}$	***σ***	$\delta_{e}/\delta_{m}$ **(%/%)**	$k_{t}/k_{p}$ **(%/%)**	
BHT5–SS	5282	2.79/90.31	845/1088	0.21	2/0.6	47/33	
BHT5–SG	5375	2.89/85.79	659/1027	0.32	6/0.7	53/34	
NAVYIII	7550	2.5/118	600/1110	0.30	0.3/0.2	49/54	
NAVYII	7700	–	914/1803	0.39	1.7/1.1	47/59	
**Samples**	$S_{11}^{E}$ **(pm^2^/N)**	$S_{12}^{E}$ **(pm^2^/N)**	$S_{13}^{E}$ **(pm^2^/N)**	$S_{33}^{E}$ **(pm^2^/N)**	$d_{33}$ **(pC/N)**	$d_{13}$ **(pC/N)**	$T_{c}$ **(°C)**
BHT5–SS	11.52	−2.51	−6.74	41.81	300	−69.39	100
BHT5–SG	11.85	−3.23	−4.74	21.12	263	−68.22	100
NAVYIII	12.00	−3.63	−4.99	13.70	219	−112	320
NAVYII	16.95	−6.60	−8.61	23.20	425	−170	350

***ρ***: density; $f_{0}^{t;p}$: fundamental resonant frequencies of the thickness (*t*) and radial mode (*p*); $\varepsilon_{33r}^{S;T}=\varepsilon_{33}^{S;T}/\varepsilon_{0}$: relative permittivity constant at constant stress (*T*) and strain (*S*), $\varepsilon_{0}$: vacuum permittivity (8.854 × 10^−12^ F.m^−1^); ***σ***: Poisson’s coefficient; $\delta_{e,m}$: electrical (*e*) and mechanical (*m*) losses; $k_{t;p}$: thickness (*t*) and radial (*p*) coupling factors; $S_{pq}^{E}$: elastic compliance at a constant electric field in *pq*–mode; $d_{pq}$: piezoelectric strain coefficient in *pq*–mode; $T_{c}$: the Curie temperature.

**Table 2:** Estimation of the energy harvesting performance of BHT5–SS, BHT5–SG, NAVYIII, and NAVYII.

Samples	$k_{13}^{2}$	FOM
BHT5–SS	4.34 × 10^−2^	5.00 × 10^−13^
BHT5–SG	4.32 × 10^−2^	5.12 × 10^−13^
NAVYIII	1.06 × 10^−1^	1.28 × 10^−12^
NAVYII	1.07 × 10^−1^	1.81 × 10^−12^

**Table 3:** Simulation results for different PEH devices according to the type of piezoelectric material used, w/ or w/o losses, under an acceleration of 10 mg^pk–pk^.

Piezoelectric Material	*F_r_*/*F_a_* (Hz/Hz)	*Z_r_*/*Z_a_* (Ω/Ω)	*P_r_*/*P_a_* (µW/µW)	*BW_r_*/*BW_a_* (Hz/Hz)
BHT5–SS w/o	156.8/158.4	5 × 10^3^/9 × 10^6^	0.1905/0.2218	0.22/0.21
BHT5–SS w/	157.0/158.2	7 × 10^4^/5 × 10^5^	0.0071/0.0067	1.92/2.15
BHT5–SG w/o	154.0/155.6	8 × 10^3^/6 × 10^6^	0.1106/0.1210	0.26/0.26
BHT5–SG w/	154.2/154.6	9 × 10^4^/2 × 10^5^	0.0057/0.0055	2.28/2.79
NAVYIII w/o	132.4/135.8	3 × 10^3^/8 × 10^7^	0.2802/0.8579	0.21/0.20
NAVYIII w/	132.4/135.8	1 × 10^4^/5 × 10^6^	0.0321/0.0288	0.59/0.73
NAVYII w/o	111.2/114.2	1 × 10^3^/7 × 10^6^	0.6774/0.1174	0.20/0.40
NAVYII w/	111.4/113.8	6 × 10^4^/6 × 10^5^	0.0067/0.0061	3.20/3.50

*Z_r_*, *P_r_*, and *BW_r_* are the optimal resistive load values, the average output power, and the half-height bandwidth at the resonance frequency *F_r_*, respectively; *Z_a_*, *P_r_*, and *BW_a_* are the optimal resistive load value, the average output power, and the half-height bandwidth at the anti-resonance frequency *F_a_*, respectively.

**Table 4:** Undamped natural frequency of the first vibration mode in short circuit conditions obtained by analytical calculation and by FE simulation for different PEH devices according to the type of piezoelectric material used (electrical and mechanical losses are not considered).

Piezoelectric Material	*ρ* (kg/m^3^)	Total Mass (g)	F^u^_r_ (Hz)		
			Analytical Model	3D FE − Model	Δ (%)
BHT5–SS	5282	2.67 × 10^−1^	156.93	158.19	0.80
BHT5–SG	5375	2.71 × 10^−1^	153.48	155.29	1.18
NAVYIII	7550	3.73 × 10^−1^	130.06	133.4	2.57
NAVYII	7700	3.80 × 10^−1^	108.41	112.09	3.39

**Table 5:** Comparison of recent piezoelectric energy harvesters’ performances (NPD: normalized volumetric power density).

Devices	Power (µW)	Acceleration (g)	Frequency (Hz)	Volume (mm^3^)	NPD (µW/mm^3^/Hz/g^2^)	Piezoelectric Material
This work (BHT5–SS *)	0.0071	0.005	157.00	49.14	3.68 × 10^−2^	BT
This work (BHT5–SG *)	0.0057	0.005	154.20	49.14	3.01 × 10^−2^	BT
Yan et al. [55]	70.000	1.000	90.000	50.00	1.56 × 10^−2^	BT
This work (NAVYIII *)	0.0321	0.005	132.40	49.14	1.97 × 10^−1^	PZT
This work (NAVYII *)	0.0056	0.005	111.40	49.14	4.09 × 10^−2^	PZT
Erturk et al. [52]	23,900	1.000	45.60	3520	1.49 × 10^−1^	PZT
Morimoto et al. [56]	5.3000	0.500	126.00	4.050	4.15 × 10^−2^	PZT
Berdy et al. [57]	118.00	0.200	49.700	588.0	1.01 × 10^−1^	PZT
Kanno et al. [58]	1.1000	1.000	1036.0	11.22	9.46 × 10^−5^	KNN
Van Minh et al. [59]	0.7310	1.000	1509.0	0.306	1.58 × 10^−3^	KNN
Won et al. [60]	3.6200	1.000	132.00	2.010	1.36 × 10^−2^	KNN
Lin et al. [61]	2970.0	1.000	357.00	1100	7.56 × 10^−3^	KNN
Wang et al. [62]	1.2500	1.000	1300.1	11.50	8.36 × 10^−5^	ZnO
Jackson et al. [63]	3.5000	0.200	149.00	30.70	1.91 × 10^−2^	AlN
Alamin Dow et al. [64]	34.780	2.000	572.00	12.76	1.19 × 10^−3^	AlN
Song et al. [65]	112.80	0.500	34.400	132.6	9.89 × 10^−2^	PVDF
Kim et al. [66]	18.560	1.750	30.000	280.0	7.21 × 10^−4^	PVDF
Montazer et al. [67]	40.900	1.000	164.00	41.19	6.05 × 10^−3^	PVDF

* with electrical and mechanical losses taken into account.

## Data Availability

Data are contained within the article.
